# Functional muscle ischemia in Duchenne and Becker muscular dystrophy

**DOI:** 10.3389/fphys.2013.00381

**Published:** 2013-12-18

**Authors:** Gail D. Thomas

**Affiliations:** Heart and Vascular Institute, Penn State College of MedicineHershey, PA, USA

**Keywords:** Duchenne muscular dystrophy, neuronal nitric oxide synthase, exercise, functional sympatholysis, sympathetic vasoconstriction

## Abstract

Duchenne and Becker muscular dystrophy (DMD/BMD) comprise a spectrum of devastating X-linked muscle wasting disease for which there is no treatment. DMD/BMD is caused by mutations in the gene encoding dystrophin, a cytoskeletal protein that stabilizes the muscle membrane and also targets other proteins to the sarcolemma. Among these is the muscle-specific isoform of neuronal nitric oxide synthase (nNOSμ) which binds spectrin-like repeats within dystrophin's rod domain and the adaptor protein α-syntrophin. Dystrophin deficiency causes loss of sarcolemmal nNOSμ and reduces paracrine signaling of muscle-derived nitric oxide (NO) to the microvasculature, which renders the diseased muscle fibers susceptible to functional muscle ischemia during exercise. Repeated bouts of functional ischemia superimposed on muscle fibers already weakened by dystrophin deficiency result in use-dependent focal muscle injury. Genetic and pharmacologic strategies to boost nNOSμ-NO signaling in dystrophic muscle alleviate functional muscle ischemia and show promise as novel therapeutic interventions for the treatment of DMD/BMD.

Duchenne and Becker muscular dystrophy (DMD/BMD) comprise a spectrum of devastating X-linked muscle wasting disease accounting for over 80% of all cases of muscular dystrophy (Bushby et al., 2010a). In DMD, boys are diagnosed as toddlers and most are wheelchair bound by age 15. Death often occurs by age 20 from respiratory complications or cardiomyopathy. More patients survive to age 30 due to home ventilation and corticosteroids, which can prolong ambulation by 2–3 years, reduce risk of scoliosis, and mitigate pulmonary and cardiac decline in the second decade (Bushby et al., 2010a,b). Despite this demonstrated efficacy, more than 25% of boys with DMD are not treated with corticosteroids due to intolerable side-effects or lack of response (Bushby et al., 2010a,b). In BMD, the disease is milder and more clinically heterogenous than DMD. Muscle weakness often is first noticed in adolescence or young adulthood. Cardiac decline in BMD patients may surpass skeletal muscle decline, with death from cardiomyopathy often occurring before age 60 (Bushby et al., 2010a). There is a clear need for additional therapeutic strategies to manage the clinical challenges of DMD/BMD.

More than 25 years have passed since mutations in the *DMD* gene were discovered to cause DMD/BMD (Monaco et al., 1986; Koenig et al., 1987). Mutations resulting in the absence of a functional dystrophin protein cause DMD, whereas mutations resulting in a reduced amount or shortened dystrophin protein cause BMD. Dystrophin is a large (427 kDa) sub-sarcolemmal protein that provides a physical link between the intracellular actin cytoskeleton and the extracellular matrix (Blake et al., 2002). Loss of dystrophin destabilizes the sarcolemma, rendering the muscle fibers susceptible to injury during contraction (Petrof et al., 1993). Repeated cycles of necrosis followed by regeneration lead to satellite cell depletion and gradual replacement of muscle by fat and connective tissue, manifesting clinically as progressive muscle wasting and weakness (Blake et al., 2002). Dystrophin also is a scaffolding protein that localizes other structural and signaling proteins to the sarcolemma, forming a highly organized multimeric dystrophin-associated glycoprotein complex (DGC) (Blake et al., 2002). Dystrophin deficiency disrupts the DGC, resulting in the absence, downregulation, or mislocalization of the dystrophin-associated proteins. Thus, numerous pathogenetic mechanisms are likely activated in dystrophin-deficient muscle in response to disruption of the sarcolemmal DGC. Identifying the key mechanistic link(s) between loss of dystrophin and the clinical phenotype of DMD/BMD has been a major focus of research efforts with the ultimate goal of discovering new therapeutic targets to slow or prevent the dystrophic process.

## The vascular hypothesis of DMD

One putative mechanism that has received increased attention recently is muscle ischemia. One of the earliest histological changes seen in dystrophic muscle, even before the onset of significant muscle weakness, is the appearance of small random groups of muscle fibers at the same stage of necrosis or regeneration surrounded by histologically normal muscle fibers (Engel, 1967). In the pre-dystrophin era, it was proposed that this characteristic focal necrosis might reflect local microvascular ischemia. The idea was that vascular insufficiency at the level of the microcirculation caused selective infarction of only those muscle fibers supplied by the obstructed blood vessels, while nearby fibers supplied by unobstructed vessels were not affected (Bramwell, 1925; Demos and Escoiffier, 1957; Cazzato, 1968). Initial experimental support for this vascular hypothesis came from studies performed more than 40 years ago in which the characteristic focal lesions of DMD muscle were reproduced in the muscles of healthy animals by occlusion of intramuscular arterioles with dextran beads, or by functional ischemia induced by a combination of arterial ligation and vasoconstrictor injection (Hathaway et al., 1970; Mendell et al., 1971, 1972). However, subsequent morphological studies did not reveal any fixed anatomical abnormalities in the skeletal muscle microcirculation of DMD patients, with the exception of replication of the capillary basal lamina (Jerusalem et al., 1974; Musch et al., 1975; Koehler, 1977; Leinonen et al., 1979). In addition, findings from studies of skeletal muscle blood flow in DMD patients were equivocal, indicating that blood flow to resting muscle was decreased, increased, or normal (Demos, 1961; Emery and Schelling, 1965; Kapuscinska et al., 1970; Paulson et al., 1974; Bradley et al., 1975; Leinonen et al., 1979). The lack of any identifiable cause of muscle ischemia in DMD patients diminished enthusiasm for the vascular hypothesis.

## Functional muscle ischemia due to loss of sarcolemmal nNOSμ

**Figure 1:**
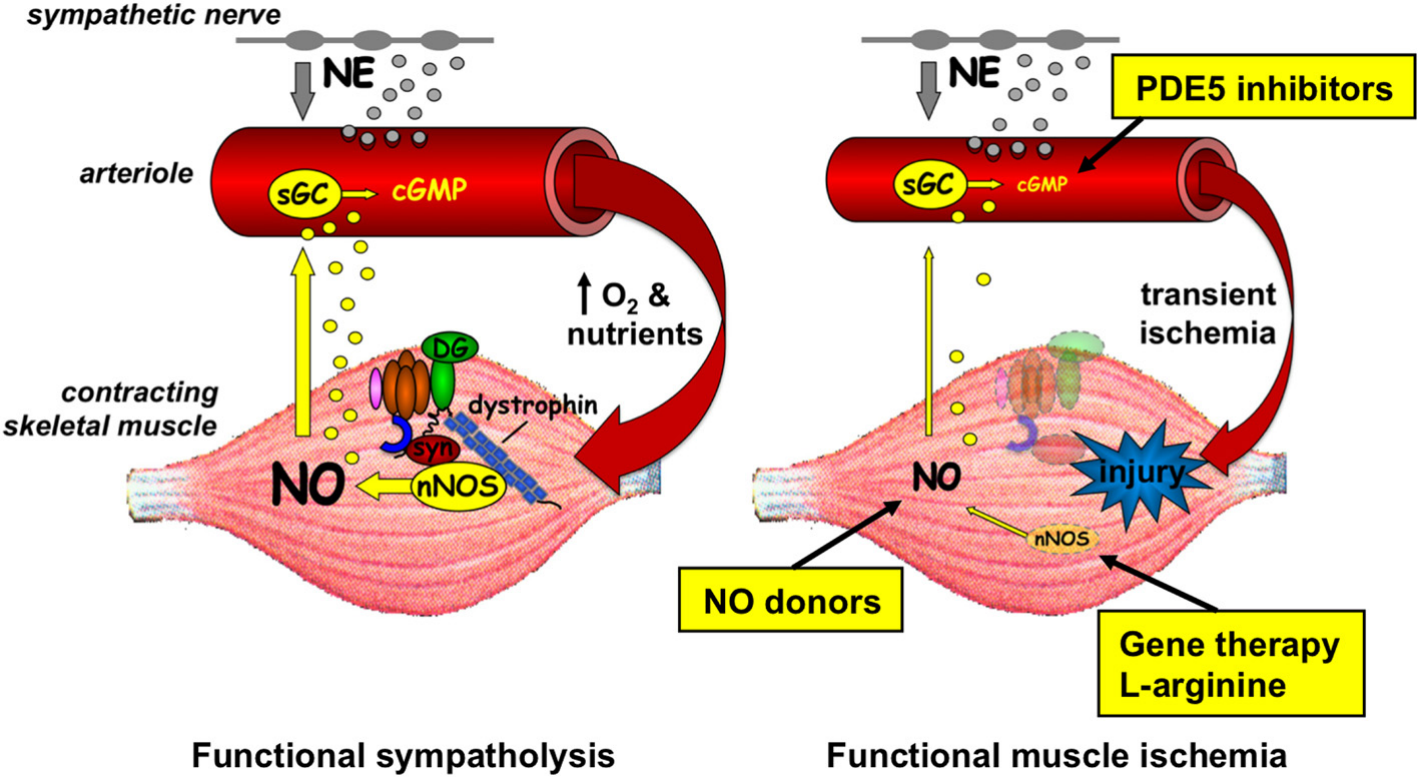
**Left panel**, in healthy muscle, nNOSμ is localized to the sarcolemma by association with the rod domain of dystrophin and the PDZ domain of α-syntrophin (syn). During muscle contraction, some of the nNOSμ-derived nitric oxide (NO) diffuses to the nearby microvessels where it increases cGMP and attenuates norepinephrine (NE)-mediated vasoconstriction. This NO-mediated functional sympatholysis optimizes blood flow in the working muscles. **Right panel**, in dystrophic muscle, nNOSμ is reduced in amount and mislocalized from the sarcolemma to the cytosol. Less NO is generated during muscle contraction, resulting in unrestrained sympathetic vasoconstriction and transient functional muscle ischemia, which may exacerbate injury of the diseased muscle fibers. Genetic and pharmacologic interventions targeting different aspects of nNOSμ-NO signaling that alleviate functional muscle ischemia in preclinical studies are shown. sGC, soluble guanylyl cyclase; DG, dystroglycan; PDE, phosphodiesterase.

It was not until after the discovery of dystrophin and the DGC that the vascular hypothesis re-emerged. Among the dystrophin-associated proteins is neuronal nitric oxide synthase μ (nNOSμ), a muscle-specific splice variant of NOS, which is recruited to the sarcolemma by two independent interactions involving its PDZ (post-synaptic density 95, discs large, and zonula occludens-1) domain. The nNOSμ PDZ β-finger interacts with the PDZ domain of the adaptor protein α-syntrophin, which binds to dystrophin's C-terminal domain, while the nNOSμ PDZ groove interacts with spectrin-like repeats 16 and 17 (R16/17) within dystrophin's rod domain (Brenman et al., 1995; Chang et al., 1996; Lai et al., 2009, 2013). Dystrophin deficiency causes nNOSμ to be misplaced from the sarcolemma to the cytosol where the residual amount of protein is also greatly reduced (Brenman et al., 1995; Chang et al., 1996; Chao et al., 1996; Torelli et al., 2004; Kobayashi et al., 2008). Parallel translational experiments in mouse models and boys with DMD showed that loss of sarcolemmal nNOSμ renders the dystrophin-deficient muscle fibers susceptible to muscle ischemia during exercise (Thomas et al., 1998, 2003; Sander et al., 2000; Lai et al., 2009). As shown in Figure 1, NO produced by sarcolemmal nNOSμ normally acts as a local paracrine signal that optimizes blood flow in the working muscles by attenuating sympathetic (i.e., α-adrenergic) vasoconstriction (Thomas and Victor, 1998; Thomas et al., 1998, 2003; Sander et al., 2000; Chavoshan et al., 2002). This protective mechanism, termed functional sympatholysis, is impaired in the mdx mouse model of DMD, in boys with DMD, and in men with BMD, resulting in functional muscle ischemia due to unopposed sympathetic vasoconstriction (Thomas et al., 1998; Sander et al., 2000; Martin et al., 2012). A similar impairment is present in the contracting muscles of nNOS null mice, indicating that the defective vasoregulation in dystrophin-deficient muscle is likely due to the loss of sarcolemmal nNOSμ (Thomas et al., 1998). This is further substantiated by proof-of-concept experiments in α-syntrophin null mice and in transgenic mice expressing a mutated α-syntrophin lacking its PDZ domain, both of which are characterized by a selective reduction of sarcolemmal, but not cytosolic, nNOSμ (Kameya et al., 1999; Adams et al., 2001; Thomas et al., 2003). These mice also display an impaired NO-dependent modulation of α-adrenergic vasoconstriction in contracting skeletal muscle, a defect that is corrected by the reestablishment of normal levels of sarcolemmal nNOSμ in transgenic mice expressing full-length α-syntrophin (Thomas et al., 2003). Furthermore, in mdx mice functional muscle ischemia is alleviated and exercise tolerance is improved by expression of a R16/17-containing synthetic dystrophin minigene that restores sarcolemmal nNOSμ (Lai et al., 2009; Zhang et al., 2013). In contrast, muscle ischemia persists in mdx mice expressing a R16/17-lacking dystrophin minigene that restores all of the DGC except nNOSμ (Lai et al., 2009).

Collectively, these observations in numerous mouse models and in DMD/BMD patients have advanced the concept that NO produced by sarcolemmal nNOSμ helps to regulate blood flow in active muscle by blunting the potentially deleterious effect of sympathetic activation during exercise. The loss of this protective mechanism may leave dystrophin-deficient skeletal muscle vulnerable to recurrent bouts of ischemia, thereby promoting muscle fatigue and exacerbating use-dependent muscle injury (Rando, 2001; Asai et al., 2007). Consistent with this hypothesis, mdx mice display an exaggerated exercise-induced fatigue response that is associated with reduced muscle perfusion and increased focal constriction of muscle microvessels (Kobayashi et al., 2008). The exaggerated fatigue response in the mdx mice appears to be due to the loss of sarcolemmal nNOSμ, as excessive fatigue also is observed in nNOS null mice and in wild type mice treated with a nNOS-specific inhibitor (Kobayashi et al., 2008). Reduced NO signaling also contributes to contraction-induced injury of dystrophin-deficient muscle. Mdx muscle exhibits progressive damage post-contraction that can be alleviated by treatment with a NO donor (Asai et al., 2007). Thus, the updated version of the vascular hypothesis of DMD suggests that the nNOSμ-NO pathway could be considered as a potential new drug target to slow disease progression in DMD/BMD patients.

However, many questions remain about the nature of the ischemic stimulus and the long term effects of ischemia on dystrophic muscle. For example, vasoconstrictor responses to activation of sympathetic nerves are reduced in the resting cremaster muscles of mdx mice (Bagher et al., 2011), suggesting that there may be less sympathetic vasoconstriction to oppose during exercise. While this does not appear to be the case in the forearm muscles of DMD/BMD patients (Sander et al., 2000; Martin et al., 2012), studies of other muscles including locomotor muscles are warranted. Whether other metabolic vasodilator pathways independent of nNOSμ-NO (e.g., H^+^, K^+^, ATP) are impaired and contribute to functional ischemia has yet to be explored in dystrophic muscle. Also unknown is if functional ischemia manifests primarily as maldistribution of blood flow within the active muscles or as reduced total muscle blood flow. Compensatory mechanisms such as angiogenesis that are engaged to counteract muscle ischemia appear to be robust in young mdx mice (Straino et al., 2004), but impaired in older mdx mice (Palladino et al., 2013), suggesting that the impact of muscle ischemia on disease progression may vary over time.

## Restoring nNOSμ-no signaling to alleviate muscle ischemia

In the absence of dystrophin, nNOSμ is both reduced in content and misplaced from the sarcolemma to the cytosol (Brenman et al., 1995; Chang et al., 1996; Chao et al., 1996; Torelli et al., 2004; Kobayashi et al., 2008). Feasible approaches to improve NO signaling in dystrophic muscle have therefore focused largely on providing exogenous NO, restoring normal levels and sarcolemmal localization of nNOSμ, or enhancing effects of residual cytosolic nNOSμ. Multiple genetic and pharmacologic strategies that boost nNOSμ-NO signaling have been shown to ameliorate many features of the dystrophic phenotype—at least in the mdx mouse. These strategies include genetic overexpression of NOS isoforms in muscle (Wehling et al., 2001; Tidball and Wehling-Henricks, 2004; Wehling-Henricks et al., 2005; Colussi et al., 2008), expression of dystrophin mini-genes that restore sarcolemmal nNOSμ (Lai et al., 2009, 2013), supplementation with the NOS substrate L-arginine to increase endogenous NO production from cytosolic nNOSμ (Barton et al., 2005; Voisin et al., 2005; Archer et al., 2006; Hnia et al., 2008; Guerron et al., 2010), treatment with NO-donating drugs (Marques et al., 2005; Brunelli et al., 2007; Benabdellah et al., 2009; Mizunoya et al., 2011; Sciorati et al., 2011; Thomas et al., 2012), and phosphodiesterase 5A (PDE5A) inhibition to prolong the half-life of cGMP arising from the residual NO generated by cytosolic nNOSμ (Asai et al., 2007; Kobayashi et al., 2008).

There is evidence to suggest that some of the beneficial effects of boosting nNOSμ-NO signaling in dystrophin-deficient muscle are likely due to correction of the abnormal vascular phenotype of functional muscle ischemia. In the mdx mouse, PDE5A inhibitors have been shown to ameliorate muscle ischemia, muscle injury, and muscle fatigue after a brief bout of downhill exercise (Kobayashi et al., 2008). In this seminal study by Kobayashi and colleagues, the effect was dramatic and immediate and was seen with two different PDE5A inhibitors, tadalafil and sildenafil. Acute tadalafil treatment has also been shown to reduce contraction-induced injury in mdx mice, while chronic treatment begun *in utero* improves muscle morphology, attenuates sarcolemmal damage, and reduces muscle fibrosis (Asai et al., 2007). Chronic sildenafil treatment has been shown to improve cardiac dynamics in mdx mice and to rescue dystrophic skeletal muscle and prolong survival in a zebrafish model of DMD (Khairallah et al., 2008; Adamo et al., 2010; Kawahara et al., 2011).

PDE5A inhibitors have proven to be safe and effective not only for patients with erectile dysfunction but also for those with pulmonary hypertension (Ravipati et al., 2007). Given the encouraging findings of pre-clinical studies in the mdx mouse, Martin and colleagues recently tested the translational potential of PDE5A inhibition to benefit patients with muscular dystrophy (Martin et al., 2012). They showed that treatment with a single dose of tadalafil rescued the abnormal vascular phenotype in the muscle of patients with BMD, fully restoring NO-dependent modulation of reflex sympathetic vasoconstriction in the exercising muscles. Tadalafil alleviated microvascular ischemia and fully restored muscle blood flow regulation in 8 of the 9 BMD patients studied (Martin et al., 2012). This finding is consistent with the hypothesis that PDE5A inhibition boosts a residual NO-cGMP signal arising from nNOSμ misplaced to the cytosol of dystrophin-deficient muscle. It is not clear why one patient did not respond to tadalafil, but conceivably some patients with DMD/BMD might have such a low level of residual muscle nNOSμ that the NO-cGMP signal is insufficient for the PDE5A inhibitor to boost. For such patients, NO-donating drugs could offer an alternative approach to restore NO signaling.

The use of NO-donating drugs in muscular dystrophy was first suggested in 2005 when Voisin and colleagues showed that 6 weeks of daily molsidomine treatment of mdx mice increased muscle utrophin, reduced creatine kinase, and reduced muscle necrosis (Voisin et al., 2005). Despite the beneficial effects observed in this short-term study, molsidomine may not be the best choice for long-term NO replacement in dystrophic muscle because the drug is metabolized to SIN-1 in the liver, which decomposes into NO and superoxide that can combine to form the damaging free radical peroxynitrite (Singh et al., 1999). Over time, this could be detrimental as dystrophin-deficient muscle cells are highly sensitive to free radical injury (Rando, 2002). Furthermore, unlike the PDE5A inhibitors, acute treatment with molsidomine did not reduce exercise-induced fatigue in mdx mice (Kobayashi et al., 2008).

Another class of NO-donating drug that has shown potential in pre-clinical mdx mouse studies is the cyclooxygenase (COX)-inhibiting NO donor (CINOD) that combines a standard COX inhibitor with a NO-donating moiety to produce dual pharmacological action (Keeble and Moore, 2002; Wallace et al., 2009). Chronic treatment (6–12 months) of mdx and α-sarcoglycan null mice with the CINOD HCT 1026, a NO-donating flurbiprofen, has been shown to improve muscle morphology and reduce muscle necrosis, inflammation, and fatigue (Brunelli et al., 2007). Similar effects have been observed in α-sarcoglycan null mice treated with NCX 320, a NO-donating ibuprofen (Sciorati et al., 2011). Improved muscle blood flow may contribute to the beneficial effects of these drugs as short-term treatment for as little as 1 month with HCT 1026 reverses functional muscle ischemia in mdx mice (Thomas et al., 2012). Importantly, the effect of HCT 1026 to prevent functional muscle ischemia does not abate with prolonged treatment, negating the concern that tolerance to the drug might develop with chronic use (Thomas et al., 2012). This is a potential drawback for some commonly used NO donors such as the organic nitrates (e.g., nitroglycerin), which lose vasodilator potency during continuous exposure (Munzel et al., 2005). Translational studies to determine if CINODs ameliorate functional muscle ischemia have yet to be carried out in patients with DMD/BMD. However, an initial 12-month pilot study has shown that co-administration of the NO donor isosorbide dinitrate and COX inhibitor ibuprofen is safe and well-tolerated in adult dystrophic patients (D'Angelo et al., 2012).

While boosting nNOSμ-NO signaling appears to be an effective means to normalize muscle blood flow regulation in the mdx mouse, it is likely to have additional mechanistic influences as well. NO derived from nNOSμ has been shown to regulate force generation, muscle mass, fatigue, muscle repair from injury, oxidative stress, and inflammation (Stamler and Meissner, 2001; Powers and Jackson, 2008). Thus, boosting NO signaling may improve DMD/BMD by a variety of mechanisms that include: (a) improving muscle blood flow and oxygen delivery (Thomas et al., 1998; Sander et al., 2000; Asai et al., 2007; Kobayashi et al., 2008; Martin et al., 2012); (b) decreasing muscle inflammation (Brunelli et al., 2007; Lai et al., 2009; Sciorati et al., 2011); (c) inhibiting histone deacetylase (HDAC) activity (Colussi et al., 2008; Cacchiarelli et al., 2010); (d) reducing muscle fibrosis (Asai et al., 2007; Guerron et al., 2010); (e) enhancing satellite cell activation during muscle repair (Anderson, 2000; Brunelli et al., 2007); (f) upregulating muscle utrophin—a dystrophin homolog with functional similarities (Voisin et al., 2005; Hnia et al., 2008); and (g) ameliorating cardiac dysfunction (Khairallah et al., 2008; Adamo et al., 2010). Although the majority of studies to date have reported favorable effects of boosting NO signaling in dystrophic muscle, further careful analysis of the long-term effects of these strategies is necessary as several studies have shown detrimental effects of mislocalized NO signaling in skeletal muscle. Increased cytosolic nNOSμ is postulated to mediate disuse muscle atrophy in normal mouse muscle by non-hemodynamic mechanisms (e.g., involving Foxo transcription factors) (Suzuki et al., 2007) and to reduce force production in dystrophic mouse muscle by hypernitrosylation of ryanodine receptors (Li et al., 2011).

## Summary

Recent findings from preclinical studies in the mdx mouse and clinical studies in DMD/BMD patients have led to renewed interest in the vascular hypothesis of muscular dystrophy. The refined hypothesis now provides a molecular mechanism, loss of sarcolemmal nNOSμ and reduced paracrine NO signaling to the muscle microvasculature, to explain the occurrence of functional muscle ischemia in dystrophin-deficient muscle. Strategies to restore nNOSμ-NO signaling in dystrophic muscle that alleviate functional muscle ischemia include dystrophin minigene therapy, PDE5A inhibition, and NO-donating drugs. The initial reports showing largely beneficial effects of restoring NO signaling in dystrophic mouse muscle suggest that reducing muscle ischemia may allow DMD/BMD patients to perform more work with less muscle injury and fatigue, thereby slowing disease progression and improving quality of life.

### Conflict of interest statement

The author has received funding and research materials from Nicox Research Institute, which is the source of one of the drugs (HCT 1026) discussed in the article.
